# Clinical Correlation of Wnt2 and COL8A1 With Colon Adenocarcinoma Prognosis

**DOI:** 10.3389/fonc.2020.01504

**Published:** 2020-08-28

**Authors:** Lihua Zhang, Xin Jiang, Yan Li, Qianqian Fan, Hongjuan Li, Linfang Jin, Liqi Li, Yufen Jin, Ting Zhang, Yong Mao, Dong Hua

**Affiliations:** ^1^Department of Oncology, Affiliated Hospital of Jiangnan University, Wuxi, China; ^2^School of Pharmaceutical Sciences, Jiangnan University, Wuxi, China; ^3^Department of Laboratory Medicine, Affiliated Hospital of Jining Medical University, Jining, China; ^4^Wuxi Medical College, Jiangnan University, Wuxi, China; ^5^Department of Pharmacy, Maternal and Child Health Hospital of Zaozhuang, Zaozhuang, China; ^6^Department of Gynecology, Maternal and Child Health Hospital of Zaozhuang, Zaozhuang, China; ^7^Department of Pathology, Affiliated Hospital of Jiangnan University, Wuxi, China; ^8^Department of Thyroid Breast Surgery, Affiliated Hospital of Jiangnan University, Wuxi, China; ^9^Institute of Cancer, Affiliated Hospital of Jiangnan University, Wuxi, China

**Keywords:** Wnt2, bioinformatics analysis, collagen type VIII alpha 1 chain, colon adenocarcinoma, prognosis

## Abstract

Wnt2 mRNA is widely expressed in various tumor tissues. Wnt2 overexpression promotes tumor growth, migration, invasion, and metastasis. However, its underlying molecular action mechanisms and clinical implications in colon adenocarcinoma (COAD) remain unclear. mRNA expression data, obtained from tissue samples, and pathophysiological data of 368 COAD patients were obtained from the Cancer Genome Atlas (TCGA) database. Further, Pearson's correlation analysis was performed to explore the correlation between the expression levels of Wnt2 and other genes in the human genome. Subsequently, a protein-protein interaction (PPI) network was constructed for hub gene identification. Overall survival and significance were determined by Kaplan-Meier analysis, and the log-rank test was used to further identify genes with prognostic significance in COAD from GEO datasets (GSE17538 and GSE39582). Subsequently, 158 tissue samples from Affiliated Hospital of Jiangnan University were used for expression verification. Gene set enrichment analysis (GSEA) was performed on high and low Wnt2 expression datasets to identify potential signaling pathways activated in COAD. In all, 10 hub genes associated with Wnt2 were screened by Pearson's correlation analysis and PPI network, with Wnt2 and COL8A1 having significantly poor prognosis by Kaplan-Meier analysis and log-rank test. Furthermore, high expressions of COL8A1 and Wnt2 were associated with poor survival both in TCGA and GEO cohorts. We further found a correlation between the expressions of Wnt2 and COL8A1 in COAD as per immunohistochemical analysis. To further elucidate the underlying molecular mechanisms of Wnt2 in COAD, we searched for pathways enriched in Wnt2 overexpressing datasets by GSEA. Our findings revealed that high Wnt2 levels were significantly associated with extracellular matrix receptor and focal adhesion pathways. Wnt2 expression correlated with COL8A1 expression in COAD; patients with high Wnt2 and COL8A1 expressions had worse survival outcomes. Pathways identified in this study prompt the molecular role of Wnt2 in COAD and provide directions to further elucidate the involved molecular mechanisms in COAD.

## Introduction

Colon cancer is a leading cause of cancer-related morbidity and mortality worldwide. It is the third most common cancer in men and the second most common cancer in women. Further, the number of newly diagnosed colon cancer cases was more than 1.8 million and the number of colon cancer-related deaths worldwide was 881000 in 2018 as per the latest GLOBOCAN data (1). Studies show that the 5-year survival rate of individuals with COAD is 65% in the United States, with 5-year survival rate of stage I and II COAD patients ranging from 80 to 90%, whereas that of patients with stage III and IV metastatic diseases being 60–71 and 8–13%, respectively. Further, Fang et al. have reported 5-year survival rates of 74.3% for stage I, 76.6% for stage II, 56.6% for stage III, and only 16.7% for stage IV (2, 3). Accordingly, early and timely diagnosis and treatment of colon cancer are very important for favorable patient prognosis. However, the diagnosis rate of colon cancer and the outcomes of comprehensive treatments, including surgery, radiotherapy, and chemotherapy, are poor, with poor 3- and 5-year survival rates. Thus, potentially effective prognostic and predictive molecular biomarkers and therapeutic indicators are particularly needed for improving patient prognosis and long-term quality of life.

The Wnt2 gene is an important member of the Wnt family. It is located on the human chromosome at 7q31 and plays an important role in cancer development. Wnt2 is highly expressed in the lungs and placenta of human fetuses, but is rarely detected in the normal gastrointestinal tract (4). Wnt2 mRNA overexpression has been reported in fibroadenomas (5), breast cancers (6), and colorectal carcinomas (7). Wnt2 affects the invasion and migration of cancer cells, proliferation, apoptosis, angiogenesis, and other adverse consequences. In previous studies, Wnt2 gene expression has been verified to be significantly high in the stroma of colorectal carcinoma patients compared with that in the stroma of normal patients (8); moreover, it promotes cancer cell metastasis and invasion through the Wnt signaling pathway. Further, injection of breast epithelial cells ectopically expressing Wnt2 into mice resulted in hyperplastic tumor development with significant fibrosis (9). In human colon cancer, Wnt2 is selectively elevated in cancer-associated fibroblasts (CAFs), resulting in increased invasion and metastasis (10). Moreover, in glioblastoma, Wnt2 regulates glioblastoma cell growth and aerobic glycolysis (11).

Collagen type VIII is a member of the short-chain collagen family, comprising COL8A1 and COL8A2, mainly produced in the cornea, aortic endothelial cells, and intramolecular mesangial cells (MCs) (12). Type VIII collagen trimer composition is variable; it may consist of two COL8A1 and one COL8A2 heterotrimers (13) or three COL8A1 homotrimers (14). Studies have shown that COL8A1 promotes the proliferation and migration of smooth muscle cells (15), and COL8A1 expression in hepatic cancer cells promotes the proliferation and invasion of Hca-F cells with a high potential of lymphatic metastasis (16). Further, loss of function of COL8A1 during embryogenesis leads to spinal fusion and scoliosis in adults (17). Moreover, COL8A1 can promote the proliferation of myogenic satellite cells, thus affecting muscle development (18).

To the best of our knowledge, no studies have been conducted on the association of Wnt2 with COL8A1, and the molecular mechanisms underlying their role and clinical significance in COAD remain unclear. In this study, we downloaded data concerning mRNA Wnt2 expression in tissue samples of 368 COAD patients from TCGA database. We further performed Pearson's correlation analysis to explore the correlation between the expressions of Wnt2 and other genes and screened the genes associated with Wnt2. We further uploaded meaningful genes and constructed a protein-protein interaction (PPI) network to search for correlations between hub genes and Wnt2. Further, to explore the prognostic significance of hub genes and Wnt2 in COAD, overall survival curves were created by Kaplan-Meier survival analysis and log-rank test, which further verified the prognostic value.

## Materials and Methods

### Patients and Tissue Samples

In the validation cohort, 158 tissue samples collected from COAD patients who underwent surgery at the Affiliated Hospital of the Jiangnan University (Wuxi, China) in 2011 were included. No patient underwent chemoradiotherapy before surgery. After harvesting, the tissues were immediately fixed in formalin and embedded in paraffin for further analysis. As per the guidelines of the American joint committee on cancer (AJCC), three pathologists determined tumor staging and were blinded to patient data. Written informed consent was provided by all participating clinicians and patients. This study was approved by the ethics committee of the Affiliated Hospital of Jiangnan University (Wuxi, China).

### mRNA Data and Correlation Analysis

mRNA expression data of tissue samples and clinical materials of 368 COAD patients were obtained from the Cancer Genome Atlas (TCGA) database. mRNA expression data of tissue samples and follow-up information were downloaded from GEO datasets (GSE17538 and GSE39582). Correlation between the expression of Wnt2 and that of other genes was analyzed using the Pearson correlation analysis. A Pearson correlation coefficient ranging between 0.5 and 1 indicated a strong correlation. *P* < 0.05 was considered statistically significant.

### Identification of Survival-Associated Hub Genes

To further screen hub genes, all significant genes were uploaded. The PPI network was constructed using the String database (http://www.string-db.org) for hub gene identification (19). Genes with a node connectivity > 2 (total edges/total nodes) were defined as hub nodes in the PPI network, and Cytoscape was used for visualizing and analyzing the network (20). Common hub genes in networks were chosen as candidates for further analysis and validated. Next, a scatter map of the top 10 hub genes was created.

Overall survival and its significance were determined by Kaplan-Meier survival analysis and log-rank test. The hazards ratio (HR) with 95% confidence intervals and log-rank *P*-value were then computed.

### Immunohistochemical Analysis

The tissues were embedded in paraffin blocks and serially sectioned into 5-μm sections. Sections were dewaxed in xylene, and hydrated in an ethanol gradient. Subsequently, the sections were heated in a citrate buffer in a microwave, cooled, and then incubated overnight with an anti-Wnt2 antibody (1:100, Abcam, Hong Kong, China) or anti-COL8A1 antibody (1:50, Abcam, Massachusetts, USA) at 4°C. After incubation with a secondary antibody (1:1, GK600710, Genetech, Shanghai, China) for 1 h at room temperature, liquid DAB substrate (1:1, GK600710, Genetech, Shanghai, China) was added. Three independent pathologists reviewed the sections, and were blinded to each other. Based on the percentage of positively stained cells, sections were graded into the following five levels: 0 (≤5%), 1 (6–25%), 2 (26–50%), 3 (51–75%), or 4 (>76%). The staining intensity corresponding to levels 0, 1, 2, or 3 was considered to be negative, weak staining, moderate staining, or strong staining, respectively. Subsequently, the immunoreactive score (IRS) was calculated for each sample by multiplying the staining intensity and positivity scores. Based on the IRSs, the samples were stratified as high (scores 9–12) and low (scores 0–9) Wnt2 or COL8A1 expression groups (21).

### Gene Set Enrichment Analysis

The COAD-associated gene clusters and pathways were identified in the c2.cp.kegg.v6.2.symbols symbols. The.gmt format data were obtained from the Molecular Signatures Database (MSigDB) using gene set enrichment analysis (GSEA) version 3.0. Furthermore, enrichment analysis was performed with a random combination number of 1000 and a false discovery rate (FDR) of 0.05 as criteria for significantly enriched genes (22). The gene sets were classified into Wnt2 high- and low-expression groups as based on the median Wnt2 expression level, and the effect of Wnt2 expression was evaluated.

### Statistical Analysis

Statistical analysis was conducted using R software (version 3.5.3, http://www.Rproject.org). Univariate and multivariate analyses of the prognostic factors were performed using the Cox proportional hazard regression model. A nomogram was formulated based on the results of multivariate analysis using the rms package. The reported statistical significance levels were all two-sided, and statistical significance was set at 0.05.

## Results

### Patient Characteristics

In the primary cohort, the clinical data of 368 COAD patients (females, 170; males, 198) were downloaded from the TCGA database, and most patients were aged 60–74 years. The average time to follow-up was 12.33 years. There were 66, 147, 98, and 57 patients in Stage I, II, III, and IV, respectively. On T staging, tumors in 8 patients were found to have started to grow into the submucosa; in 65, into the muscularis propria; in 254, into the subserosa; and in 41, directly into other organs or structures. On N staging, 221 patients showed no cancerous cells in lymph nodes and 147 showed cancerous cells in 1 to 2 lymph nodes. Further, on M staging, 311 patients showed no metastasis and 57 patients showed metastasis. General patient information is summarized in Table 1.

**Table 1:** Clinical characteristics of 368 COAD patients.

**Characteristics**	**Number of cases**	
**AGE (YEARS)**		
0–44	21	5.71%
45–59	78	21.20%
60–74	155	42.12%
75–90	114	30.97%
**GENDER**		
Female	170	46.20%
Male	198	53.80%
**TNM STAGE**		
Stage I	66	17.90%
Stage II	147	39.90%
Stage III	98	26.60%
Stage IV	57	15.40%
**T STAGE**		
T1	8	2.10%
T2	65	17.60%
T3	254	69.00%
T4	41	11.10%
**N STAGE**		
N0	221	58.90%
N1	86	23.80%
N2	61	17.30%
**M STAGE**		
M0	311	76.20%
M1	57	14.10%

*TNM, Tumor-node-metastasis*.

### Screening of 10 Hub Genes Related to Wnt2

**Figure 1:**
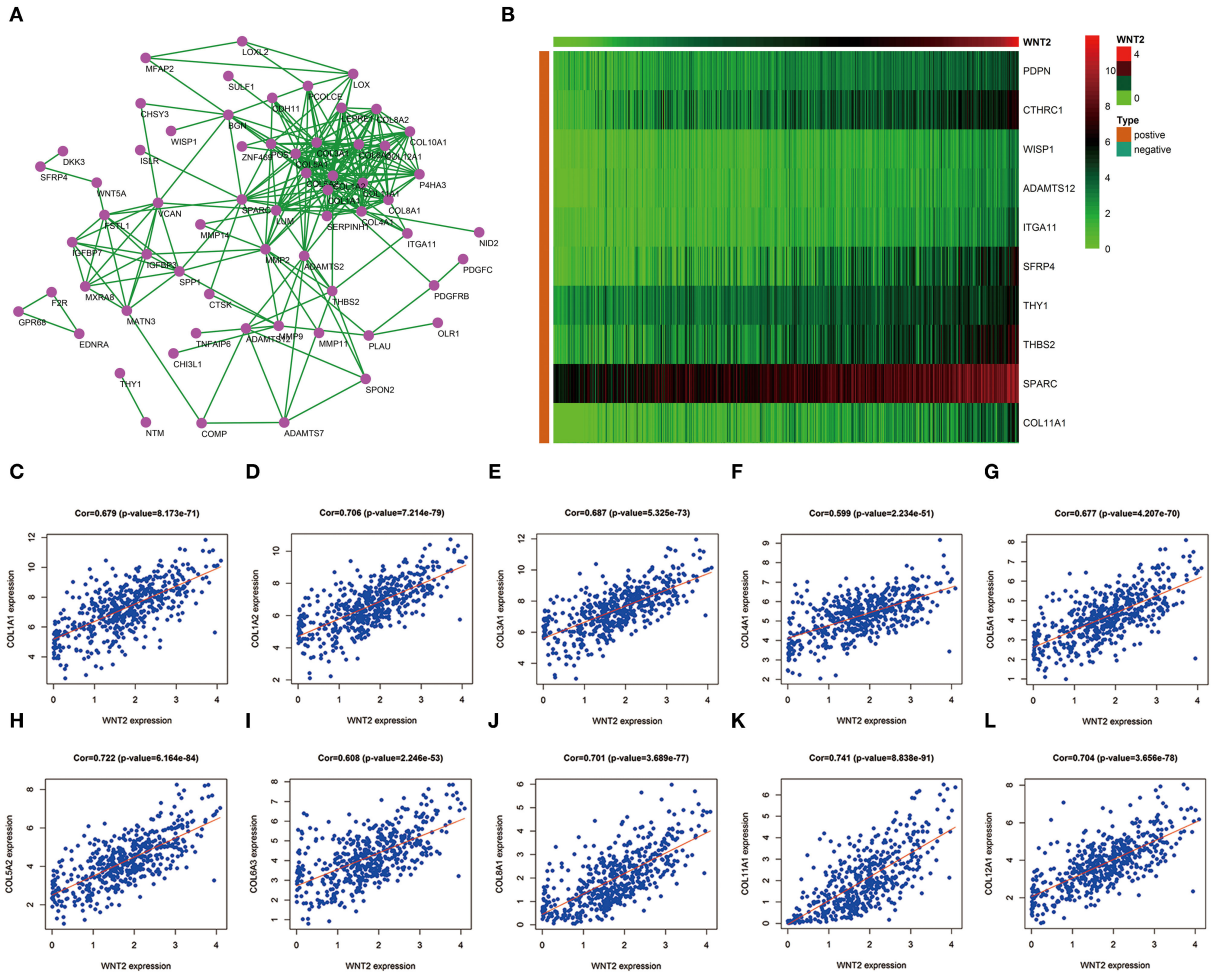
Prediction of 10 hub genes related to Wnt2 based on COAD data. **(A)** Hub genes related to Wnt2 in the protein-protein interaction (PPI) network constructed using the STRING website. **(B)** Hub genes related to Wnt2 in the heatmap. **(C)** COL1A1; **(D)** COL1A2; **(E)** COL3A1; **(F)** COL4A1; **(G)** COL5A1; **(H)** C0L5A2; **(I)** COL6A3; **(J)** COL8A1; **(K)** COL11A1; and **(L)** COL12A1.

Pearson's correlation analysis showed that 210 genes positively correlated with Wnt2 (*r* > 0.5, *P* < 0.001), whereas 3 genes negatively correlated with it (*r* < −0.5, *P* < 0.001). To further screen hub genes, the significantly expressed genes were uploaded and a PPI network diagram was constructed using the STRING website (Figure 1A). The top 10 significantly related genes were showed by heatmap (Figure 1B). Following this, Cytoscape was used to visualize PPI networks and find the hub genes. A total of 10 hub genes were identified, including COL1A1, COL1A2, COL3A1, COL4A1, COL5A1, COL5A2, COL6A3, COL8A1, COL11A1, COL12A1. In addition, the Pearson's correlation coefficients between these 10 hub genes and Wnt2 were as follows: 0.679, 0.706, 0.687, 0.599, 0.677, 0.722, 0.608, 0.701, 0.741, and 0.704, respectively (Figures 1C–L).

### Identifying Survival-Related Hub Genes

**Figure 2:**
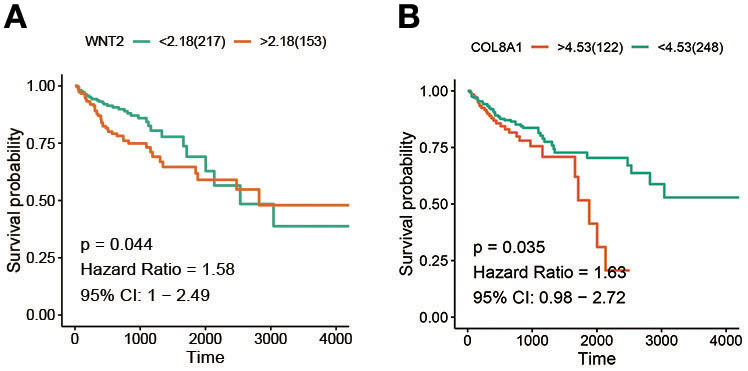
Screening of survival-related hub genes. **(A)** The relationship between the expression of Wnt2 and overall survival of colon cancer patients. **(B)** The relationship between BRAF mutation and overall survival of colon cancer patients.

For the 10 hub genes and Wnt2, overall survival and its significance were determined by Kaplan-Meier survival analysis and log-rank test. The results showed that only Wnt2 (*P* = 0.044; Figure 2A) and COL8A1 (*P* = 0.035; Figure 2B) significantly correlated with patient prognosis.

### High Expression Levels of COL8A1 and Wnt2 Indicate Poor COAD Prognosis

**Figure 3:**
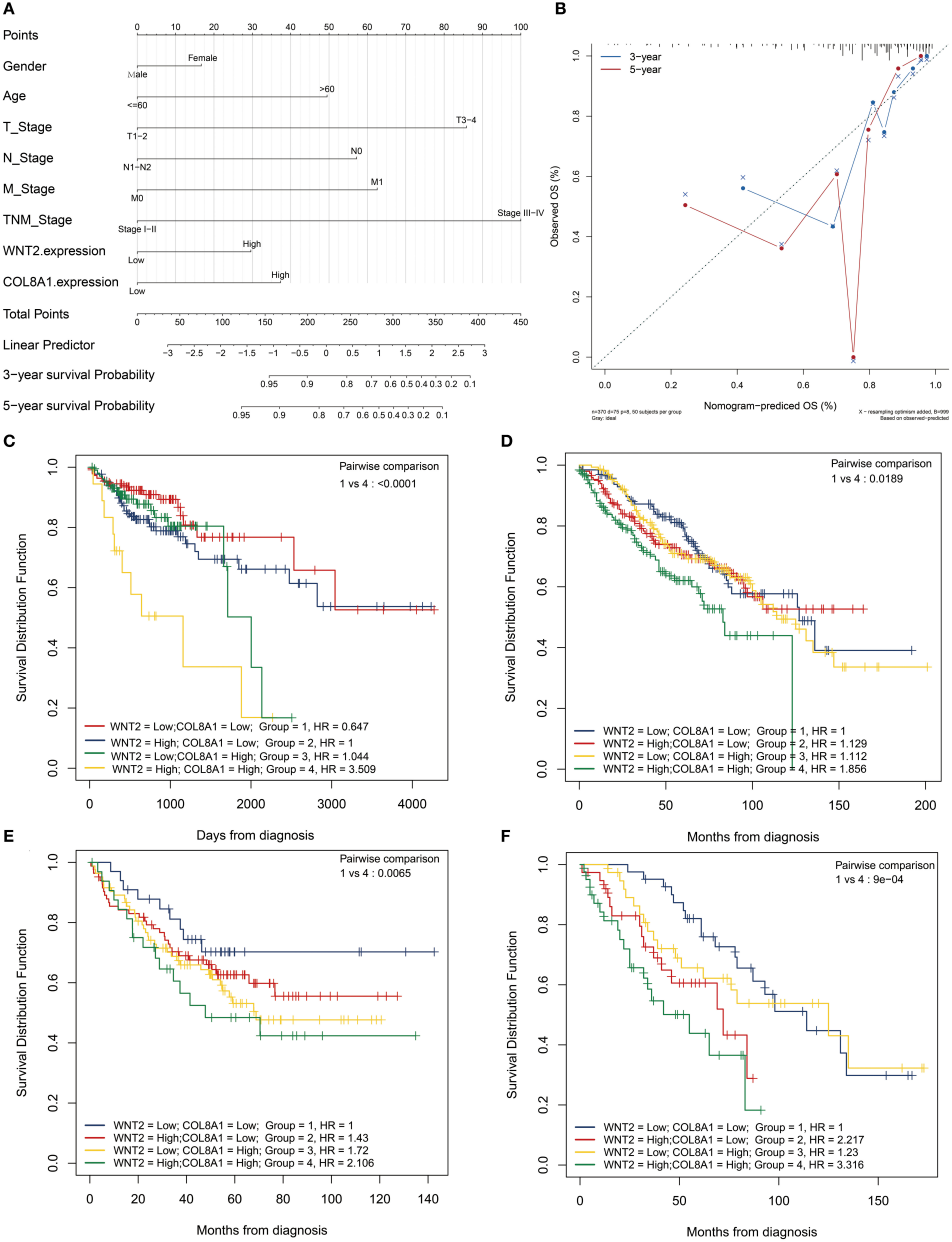
Prognostic value of Wnt2 and COL8A1 in COAD. **(A)** Prognostic nomogram for COAD based on clinical features, and Wnt2 and COL8A1 expression levels. **(B)** Calibration curves for clinical prediction model curves for the association of Wnt2-based prognostic score with clinicopathological variables. **(C)** Patients with low Wnt2 and COL8A1 expression have a better prognosis compared to patients with high Wnt2 and COL8A1 expressions in the TCGA cohort. **(D)** patients from GSE17538 cohort. **(E)** Patients from GSE39582 cohort. **(F)** Patients from Jiangnan University cohort.

The nomogram illustrated that TNM stage had the largest contribution to prognosis, followed by T stage, N stage, and age. Moreover, high expression levels of COL8A1 and Wnt2 associated with lower low survival rates (Figure 3A). Next, we tested the reliability and validity of the model, and the results indicated that calibration plots presented excellent agreement in the primary cohort, and acceptable agreement in the validation cohort between the nomogram prediction and actual observation for 3- and 5-year overall survival (OS) (Figure 3B). Thus, the survival analysis showed that patients with high expression levels of Wnt2 and COL8A1 had a poor prognosis compared with those with low expression levels of Wnt2 and COL8A1 (Figure 3C). Similar results were observed in GSE17538 and GSE39582 cohorts, thus, verifying the findings (Figures 3D,E).

**Figure 4:**
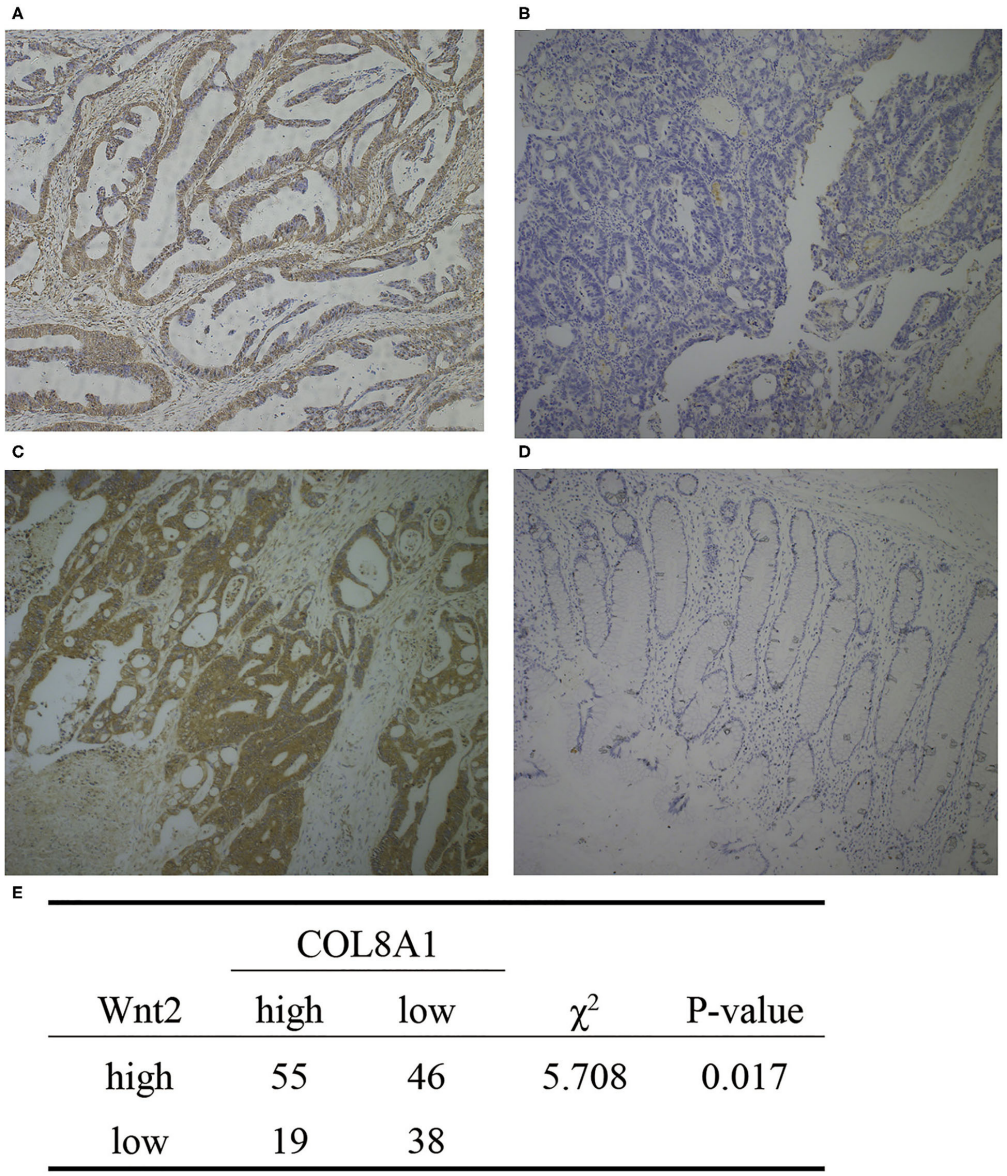
Expression of Wnt2 and COL8A1 in COAD tissues. **(A)** High expression of Wnt2. **(B)** Low expression of Wnt2. **(C)** High expression of COL8A1. **(D)** Low expression of COL8A1. **(E)** Analysis of the expression of Wnt2 and COL8A1 in 158 tissue samples. Images are taken at a magnification of 10×.

To further explore the relationship of Wnt2 and COL8A1 with survival, we evaluated the expression levels of Wnt2 and COL8A1 in the tissues of 158 COAD patients via immunohistochemical analysis. Depending on the staining intensity, the samples were divided into Wnt2 high- and low-expression groups (Figures 4A,B) and COL8A1 high and low-expression groups (Figures 4C,D). The results showed that high expression levels of Wnt2 accounted for 63.9%, whereas high expression levels of COL8A1 accounted for 46.8% of the total population. In addition, the expressions of both Wnt2 and COL8A1 were high in 55 tissues and low in 38 tissues. The expression levels of the two molecules were correlated (*P* = 0.017, Figure 4E). The survival analysis also showed that patients with high expression levels of Wnt2 and COL8A1 had a poor prognosis compared with those with low expression levels of Wnt2 and COL8A1 (Figure 3F). We then analyzed the factors of poor prognosis by univariate and multivariate cox proportional hazards analyses. In univariate overall survival (OS) analysis, we observed that COL8A1 expression (*P* = 0.017), WNT2 expression (*P* = 0.049), and WNT2 and COL8A1 expression (*P* = 0.002) retained significance as prognostic factors (Table 2). In multivariate OS analysis, we showed that WNT2 and COL8A1 (*P* = 0.018, Table 2) was an independent predictor of poor prognosis. Accordingly, our data demonstrated that high expression of WNT2 and COL8A1 could predicate a poor prognosis of patients with COAD.

**Table 2:** Univariate and multivariate COX regression analysis of the prognostic factors of COAD.

**Characteristics**	**Univariate analysis**		**Multivariate analysis**	
	**HR (95%CI)**	***P***	**HR (95%CI)**	***P***
Age (>60 vs. ≤ 60)	1.23 (0.66–2.29)	0.522		
Gender (Male vs. Female)	1.52 (0.91–2.53)	0.106		
T stage (T3-T4 vs. T1-T2)	1.18 (0.58–2.37)	0.652		
N stage (N2-N3 vs. N0-N1)	1.16 (0.66–2.03)	0.607		
M stage (M1 vs. M0)	1.47 (0.53–4.11)	0.457		
TNM stage (III-IV vs. I-II)	1.55 (0.96–2.52)	0.076		
COL8A1 expression (High vs. Low)	1.78 (1.11–2.87)	0.017	1.48 (0.9–2.44)	0.12
WNT2 expression (High vs. Low)	1.89 (1.17–3.06)	0.049	1.28 (0.72–2.28)	0.406
WNT2 and COL8A1 expression (High vs. Low)	2.81 (1.64–4.81)	0.002	2.19 (1.14–4.19)	0.018

### GSEA and Cluster Profiler Recognize the Wnt2-Related Signaling Pathway

**Figure 5:**
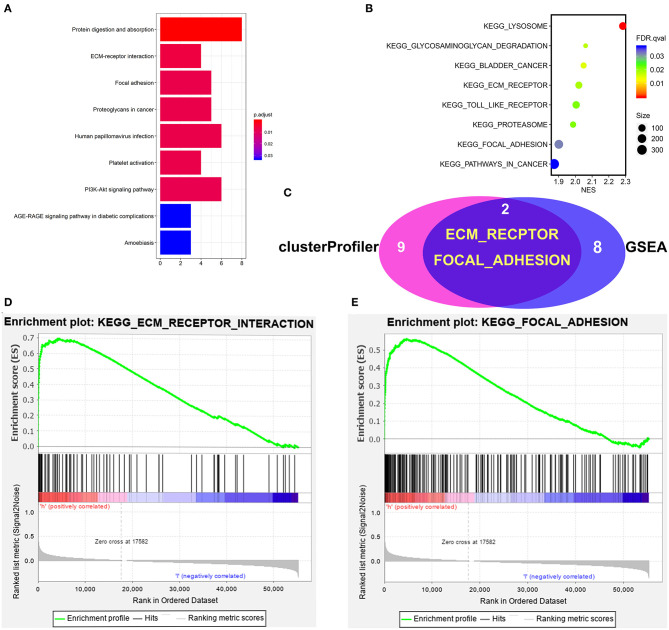
Gene set enrichment analysis of Wnt2 in COAD datasets. **(A)** ClusterProfiler analysis found nine significantly enriched signaling pathways. **(B)** GSEA analysis found eight significantly enriched signaling pathways. **(C)** The intersection of clusterProfiler analysis and GSEA analysis. **(D)** ECM-receptor pathway. **(E)** Focal adhesion pathway.

To identify signaling pathways that may be activated in COAD, GSEA and clusterProfiler analysis were performed on high and low Wnt2 expression datasets (FDR *P* < 0.05, NOM *P* < 0.05), and the most significantly enriched signaling pathways were selected based on the normalized enrichment score (NES). ClusterProfiler analysis found nine significantly enriched signaling pathways: protein digestion and absorption, ECM-receptor pathway, focal adhesion pathway, proteoglycans in cancer, human papillomavirus infection, platelet activation pathway, PI3K-Akt signaling pathway, AGE-RAGE signaling pathway in diabetic complications, and amoebiasis (Figure 5A). GSEA analysis found eight significantly enriched signaling pathways: lysosome, bladder cancer, ECM-receptor pathway, toll link receptor, proteasome, pathways in cancer, and focal adhesion pathway (Figure 5B). Both GSEA analysis and clusterProfiler analysis found that ECM-receptor pathway (Figure 5D) and focal adhesion pathway (Figure 5E) are significantly enriched signaling pathways (Figure 5C).

## Discussion

COAD is a common malignant tumor with high morbidity and mortality worldwide. The occurrence and development of cancer are affected by many factors, and different gene expressions affect cancer. Therefore, identifying new biomarkers for the diagnosis and treatment of cancer is highly significant. Previous studies have shown that Wnt2 is highly expressed in cancer cells compared with its expression in normal cells and that Wnt2 affects the invasion and migration of cancer cells, proliferation, apoptosis, angiogenesis, and other adverse consequences.

To further explore the underlying action mechanism of Wnt2 in tumors, RNA high-throughput sequencing data of COAD patients, as well as corresponding clinicopathological data were downloaded from the TCGA and GEO database. In this study, we screened 10 hub genes related to Wnt2 and found COL8A1 to be a survival-related hub gene. Subsequently, we verified the correlation between the expressions of Wnt2 and COL8A1 in 158 tumor tissues of COAD patients by immunohistochemical analysis (*P* = 0.017).

Moreover, a survival analysis of 368 patients from the TCGA database found that patients with high expression levels of Wnt2 and COL8A1 had worse outcomes, consistent with previous studies (23, 24). Overall, our findings suggested that Wnt2 and COL8A1 play an important role in the prognosis and clinical progression of COAD, and will provide a direction for the identification of molecular mechanisms involved in COAD.

This study had several limitations. First, this study was mainly based on the analysis of clinical data of COAD patients from the TCGA database, and histological verification was conducted only in 158 patients with colorectal cancer from the affiliated hospital of Jiangnan university. In the follow-up study, we plan to increase the sample size and conduct a multi-center study. Second, we searched for the Wnt2-related pathway via GSEA, and there was a lack of relevant mechanism research. Accordingly, in the future experiments, we plan to further explore the relevant mechanisms of Wnt2 and COL8A1.

## Data Availability Statement

Publicly available datasets were analyzed in this study. This data can be found here: TCGA https://portal.gdc.cancer.gov/.

## Ethics Statement

The studies involving human participants were reviewed and approved by Affiliated Hospital of Jiangnan University Institutional Review Board. The patients/participants provided their written informed consent to participate in this study.

## Author Contributions

LZ designed the project. XJ did the experiment and wrote the article. YL organized the data. LJ, TZ, and LL collected samples. YM and DH conducted the research. LZ, XJ, and YL contributed equally to this work. All authors contributed to the article and approved the submitted version.

## Conflict of Interest

The authors declare that the research was conducted in the absence of any commercial or financial relationships that could be construed as a potential conflict of interest.
